# Supplementary material for: FastqPuri: high-performance preprocessing of RNA-seq data
Source: BMC Bioinformatics. 2019 May 3;20:226. doi: 10.1186/s12859-019-2799-0 (PMC6500068; doi:10.1186/s12859-019-2799-0)
Supplement: Supplementary file 2 — Archive of FastqPuri. Archive containing all files needed to install and run FastqPuri v1.0.6. Date stamp March 22, 2019. (GZ 47,819 kb) [file 12859_2019_2799_MOESM2_ESM.gz › FastqPuri-1.0.6/html/struct__bfilter.html]

FastqPuri: \_bfilter Struct Reference


FastqPuri

Public Attributes |
List of all members

\_bfilter Struct Reference

Bloom filter structure.
More...

`#include <bloom.h>`

|  |  |
| --- | --- |
| Public Attributes | |
| int | kmersize |
|  | |
| int | hashNum |
|  | |
| int | kmersizeBytes |
|  | |
| double | falsePosRate |
|  | |
| uint64\_t | bfsizeBits |
|  | |
| uint64\_t | bfsizeBytes |
|  | |
| uint64\_t | nelem |
|  | |
| unsigned char \* | filter |
|  | |

## Detailed Description

Bloom filter structure.

## Member Data Documentation

## ◆ bfsizeBits

uint64\_t \_bfilter::bfsizeBits

bloom filter size (bits) (m)

## ◆ bfsizeBytes

uint64\_t \_bfilter::bfsizeBytes

bloom filter size (bytes)

## ◆ falsePosRate

double \_bfilter::falsePosRate

False positive rate

## ◆ filter

unsigned char\* \_bfilter::filter

filter sequence

## ◆ hashNum

int \_bfilter::hashNum

number of hash functions used to construct the filter

## ◆ kmersize

int \_bfilter::kmersize

kmer size (number of elements)

## ◆ kmersizeBytes

int \_bfilter::kmersizeBytes

Bytes needed to store the kmer (4bases ~ 1byte)

## ◆ nelem

uint64\_t \_bfilter::nelem

number of elements encoded in the bloom filter (n)

---

The documentation for this struct was generated from the following file:

- include/bloom.h


---

Generated on Mon Mar 19 2018 23:42:01 for FastqPuri by  

 1.8.14
